# Love thy neighbour: facilitation through an alternative signalling modality in plants

**DOI:** 10.1186/1472-6785-13-19

**Published:** 2013-05-07

**Authors:** Monica Gagliano, Michael Renton

**Affiliations:** 1 Centre for Evolutionary Biology, School of Animal Biology, University of Western Australia, Crawley, WA 6009, Australia; 2 School of Plant Biology, University of Western Australia, Crawley, WA 6009, Australia

**Keywords:** Facilitation, Species interactions, Signalling

## Abstract

**Background:**

Both competitive and facilitative interactions between species play a fundamental role in shaping natural communities. A recent study showed that competitive interactions between plants can be mediated by some alternative signalling channel, extending beyond those channels studied so far (i.e. chemicals, contact and light). Here, we tested whether such alternative pathway also enables facilitative interactions between neighbouring plant species. Specifically, we examined whether the presence of a ‘good’ neighbouring plant like basil positively influenced the germination of chilli seeds when all known signals were blocked. For this purpose, we used a custom-designed experimental set-up that prevented above- and below-ground contact and blocked chemical and light-mediated signals normally exchange by plants.

**Results:**

We found that seed germination was positively enhanced by the presence of a ‘good’ neighbour, even when the known signalling modalities were blocked, indicating that light, touch or chemical signals may not be indispensible for different plant species to sense each other’s presence.

**Conclusions:**

We propose that this alternative signalling modality operates as a general indicator of the presence of heterospecifics, enabling seeds to detect and identify a neighbour prior to engaging in a more finely-tuned, but potentially more costly, response.

## Background

The notion that neighbouring species negatively impact on one another through competitive interactions has long been the key component of many classic ecological theories and is still the prevailing view amongst plant ecologists. Naturally, plant neighbour relationships take several forms. While there is no doubt that competition plays a fundamental role in shaping plant communities, it has become increasingly recognised that facilitative or positive interactions are also ubiquitous [1] and the influence of facilitation is equally important in regulating the composition and diversity of communities [2-4]. Indeed, many plants are very good companions and literally facilitate each other [5,6] by improving growth [7], fixing nitrogen [8], nurse cropping [9], controlling pests [10] or attracting beneficial organisms such as insects [11] or mycorrhizae [12,13].

Much of our current understanding of the mechanisms underlying these facilitative interactions amongst plants has been primarily focused on the effects of light (and shade), chemical signalling or physical proximity and contact. For example, we know that the shade of perennial canopies may benefit young seedlings and smaller species by protecting them from temperature extremes and excessive water loss, and providing favourable soil texture and chemistry [14]. And analogously to animal systems such as the case of small abalone juveniles finding shelter amidst the spines of adult sea urchins, some plants associate with benefactor species that can physically protect them from predation by virtue of their spine-covered stems [15]. Furthermore, the recent literature is replete with examples of plants associating with benefactor species that protect them through visual or olfactory concealment by interfering with the ability of an herbivore to find and feed on them [16]. In all of these examples of facilitation, the mechanism through which the benefit is conferred from one plant to another is relatively clear and mediated through recognised pathways.

**Figure 1:**
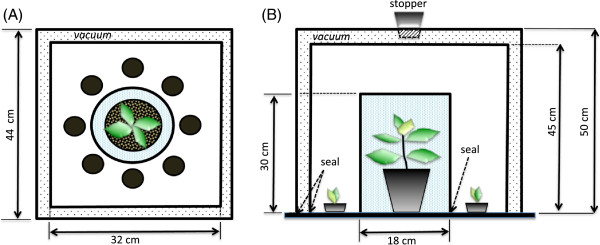
**Diagram illustrating the custom-designed experimental unit (not in scale).** (**A**) Chilli seeds were arranged in a circle around the adult plant sealed the central cylindrical box. (**B**) All seeds and adult plants within each unit were housed within 2 different sized square boxes, one inside the other, with the air in between the two boxes removed using a vacuum pump. This schematic representation has been adapted from [17].

In a recent study, we demonstrated that plants are able to sense and/or affect their neighbours and using some alternative pathway(s) beyond light, chemical signals or physical contact [17]. Specifically, we showed that seeds and seedlings of chilli plants (*Capsicum annuum*, Solanaceae) are able to discriminate between the presence of an adult conspecific and a fennel plant, known anecdotally to negatively impact on the growth of neighbouring plants through aggressive competitive interactions and hence purposely chosen as the ‘bad neighbour’. Specifically, the volatile chemicals of fennel hindered the germination rate of chilli seeds; however germination rate of chilli was accelerated in treatments where fennel was present but its signals were partially or totally blocked. This demonstrated that plants were able to sense their neighbours even when all known communication channels are blocked (i.e. light, chemicals and touch) and most importantly, recognise the potential for the interfering presence of a ‘bad neighbour’ and modify their growth accordingly. The question remains open, though, whether such alternative means of communication occur amongst ‘good neighbours’. By adopting the same experimental design described in [17], we tested whether the presence of a ‘good’ neighbouring plant could positively influence germination rates of chilli seeds when all known signals are blocked (Figure 1). For this purpose, we chose the Basil plant (*Ocimum basilicum*, Lamiaceae), because of its well-known capacity to produce a large number of secondary and organic volatiles inhibiting germination and root growth of common competitive weeds (e.g. barnyardgrass and lambsquarters; [18]) and functioning as effective natural insecticides (reviewed in [19]). Besides, gardeners commonly regard it as the ideal companion to chilli plants by virtue of its ability to keep the soil moist and act as organic living mulch. Hence, we wanted to test whether the presence of basil would positively enhance germination rates of chilli seeds, both when open contact was allowed and when all known signals were totally blocked. We found that this is the case, indicating that both competitive and facilitative interactions between plants are mediated by signalling modalities that remain to be identified.

## Results

**Figure 2:**
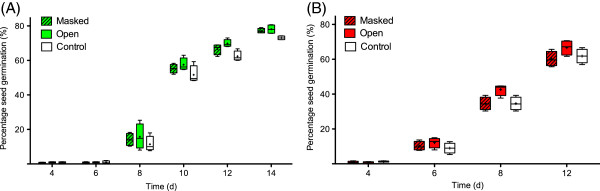
**Effects of the presence of a neighbouring (A) basil and (B) chilli plant on the percentage of chilli seeds germinating over time.** The median, inter-quartile range and 5-95 percentiles are represented by the middle bar, the top and bottom of box and the whiskers respectively. The mean is also represented by the small ‘+’ symbol.

For the *Basil neighbour experiment*, there was an overall difference between treatments (p < 0.05; Figure 2A and Additional file 1: Table S1). Germination in the MASKED and the OPEN treatment was greater than in the CONTROL treatment (p = 0.011), and germination in the MASKED treatment did not differ significantly from the OPEN treatment (p = 0.21). Germination also varied significantly over time (p < 0.0001), with generally low rates in the absence of a neighbouring plant.

In the *Chilli neighbour experiment*, there was an overall difference between treatments (p = 0.041; Figure 2B and Additional file 1: Table S2). Germination in the MASKED was not significantly different to that in the CONTROL treatment (p = 0.90) but germination in the OPEN treatment was greater than in the CONTROL treatment (p =0.011). Germination also varied significantly over time (p<0.0001), with generally low rates in the absence of a neighbouring plant.

## Discussion

The presence of basil positively enhanced germination rates of chilli seeds, validating the claims of many gardeners who recognise the beneficial effect of basil on the growth of chilli plants. Interestingly, this was true even when all known signals from the basil were blocked, indicating that the facilitative interaction observed between these two species is mediated through a signalling modality other than those studied thus far (i.e. light, chemicals and touch). Most importantly, the fact that germination of chilli seeds did not differ between the MASKED and OPEN treatment indicates that light, touch or chemical signals may be important but clearly not necessary for chilli seeds and basil plants to sense each other’s presence. The observed differences in germination between treatments were relatively small, but even small differences in germination are likely to have significant effects on competitiveness and thus on fitness. In fact, carryover effects associated with different germination responses to the surrounding environment can be responsible for a large amount of the variation in growth and reproductive performance among older individuals (e.g. [20]). Over multiple generations, such differences in competitiveness and relative fitness resulting from small differences in germination will have increasing cumulative effects on selection. From an evolutionary perspective, it could also be argued that there is likely to be some adaptive competitive advantage to these responses, for them to have evolved in the first place. Overall, our findings highlight the existence and importance of alternative signalling modalities in plants and invites further investigation on the generality and ecological role of this phenomenon.

Intriguingly, chilli seeds in the *Chilli neighbour experiment* did not appear to discriminate between the control and the presence of an adult conspecific when this was masked, but did so in the OPEN treatment when light and chemical signals, for example, were accessible. These findings are particularly significant for two reasons. Firstly, they demonstrate that signalling mediated by chemicals and light is an important modality amongst conspecifics. But most notably, they indicate that the alternative signalling channel, which does not rely on light, chemicals or touch is particularly important when the neighbouring plant is not a conspecific. Because the presence and specific identity of neighbours influence germination timing and success, the existence of an adaptive mechanism that allows a plant to detect its neighbours (and potentially its forthcoming competitive environment), and hence to regulate its developmental responses accordingly at the very onset of its life (i.e. seed stage) is clearly advantageous. By operating as a general indicator of the presence of a heterospecific, this signalling modality may indeed be such a strategy enabling seeds to detect the presence of a neighbour prior to engaging into a more fine-tuned response (which may be energetically expensive; e.g. accelerate growth) tailored to the specific identity of the neighbour (i.e. friend or foe; see also [21]).

## Conclusions

Previous authors have pointed out that facilitation can be an important interaction and one that has not received enough attention from ecologists. We further demonstrate the importance of facilitation between plant species and most interestingly, we provide the first evidence for facilitation occurring via novel communication means other than the established pathways that have already been elucidated. While no mechanistic explanation of how plants may perform the observed feat is available yet (but see [22]), it is becoming clearer that some of the underlying conditions required for such a channel to function as described here include the emission of a signal that not only propagates rapidly to convey real-time information about neighbouring plants but also can be analyzed quickly. We have previously suggested that acoustic signals may offer such a mechanism for mediating plant-plant relationships [23] and proposed that the such signals may be generated in plants by biochemical processes within the cell, where nanomechanical oscillations of various components in the cytoskeleton can produce a spectrum of vibrations [24]. The present findings further support the hypothesis of acoustic communication, and hence extends our understanding on how plants may employ this modality to selectively mediate facilitative as well as competitive interactions with surrounding heterospecific neighbours.

## Methods

During March-April 2011, chilli seeds (*Capsicum annuum*) were used for 2 experimental germination tests conducted at the Plant Growth Facilities at the University of Western Australia. In the first experiment (i.e. *Basil neighbour experiment*), 3600 chilli seeds were randomly apportioned among 3 treatments, each replicated 4 times and kept randomly interspersed throughout a 5.30 m^2^ Controlled Environment Room (CER) fitted with high-intensity discharge lamps (standard metal halide supplemented with halogen; 650 μmol of photons s^-1^ m^-2^ photosynthetic active radiation). The experimental units consisted of a group of 12 petri dishes, each one containing 25 seeds, which were sandwiched between layers of 2 mm thick felt to retain moisture and ensure darkness. Petri dishes were arranged at c.8 cm from each other and placed in a circle around an adult *O. basilicum* plant positioned in the centre of the experimental unit (see Figure 1 and detailed description in [17]). Specifically, the *O. basilicum* plant was either left open to allow both light and chemical communication to take place [OPEN treatment] or sealed in a central cylindrical box covered in black plastic to block all light wavelengths and airborne chemical signals (i.e. no volatile or water-soluble chemicals from any adult plants could affect seed germination) [MASKED treatment]. The CONTROL treatment consisted of seeds arranged around the central cylindrical box, which was left empty but covered in black plastic to account for any effects of the colour of this shield itself.

All seeds and adult plants within a replicate unit were then housed within 2 different sized square boxes (44×44×50 cm & 32×32×45 cm respectively), one inside the other, with the air in between the two boxes removed using a pump to create a vacuum and thus avoid interference between adjacent experimental units at any time. The whole experimental unit was custom-made in colourless cast acrylic material (Moden Glas), which transmitted 92% of visible light, but was opaque to ultraviolet and infrared wavelengths (Figure 1; as per [17]). Each day, all experimental units were randomly re-interspersed throughout the growth room to avoid any potential artefacts due to their position in the room. Similarly each day, individual petri dishes within each unit were randomly re-arranged in the circular configuration around the central box to avoid any potential confounding effects of their position within the experimental unit. Using an U12-011 - HOBO® Temperature/RH Data Logger, we recorded the temperature within the experimental units over 24 h for 25 consecutive days to ensure that any difference in seed germination measured between treatments was not due to differences in the temperature inside the boxes caused by the presence or absence of adult plants (No difference between temperature profiles across treatments over time; Repeated-measure ANOVA, F_46, 115_ = 1.11, P = 0.32). Seeds were kept at 18°C during the day, 13°C over night and under a 12 h light: 12 h dark cycle. Seeds were inspected and watered every 24 hrs. To avoid any potential atmospheric exchange of volatiles that could have interfered with our measurements, each experimental unit was transferred one at a time to a separate room where the 2 external square boxes were opened; all petri dishes were then removed and inspected, while the rest of the unit (including the base and the central cylindrical box) was taken outdoors and opened. This procedure was conducted to aerate the adult plants sealed in the box, but was done for all units (i.e. with or without plant in the central cylindrical box). Germination rates in each treatment were monitored and recorded every other day for 12 days, after which the number of germinating seeds reached an asymptote.

The same experimental design described above was then repeated by substituting the basil plants with adult chilli plants (i.e. *Chilli neighbour experiment*). This experiment was conducted as a control benchmark to enable us to distinguish the possible positive effect of growing next to an adult conspecific from the effect of a potentially beneficial heterospecific.

All statistical analyses were carried out in R using binomial generalised linear mixed effects models (GLMMs) with the base package and the lme4 package [25,26]. Binomial generalized linear models were used as they are the most valid means of dealing with binomial data, such as germination data, and the mixed effects versions of the models were employed as they are the most valid and powerful means of dealing with a design such as ours, which had nesting (Petri dishes within containers) and repeated measures over time [27]. A separate but similar analysis was conducted for each of the two experiments, based on standard step-wise model simplification [27]. First, a full model was fitted to the data. This contained fixed effects for treatment, time and their interaction, constant random effect for experimental container and Petri dish nested within experimental container, and continuous random time effects for experimental container and Petri dish nested within experimental container. This full model was then sequentially simplified, step-by-step, in the standard way [27]. At each step, a simplified model with one term dropped from the previous best model was compared to the previous best model using both Akaike’s Information Criterion (AIC) and a likelihood ratio chi-squared test. AIC values were computed for each of the candidate models and the model with the lowest AIC value was selected as the best model of the observed data in the standard way [28]. The likelihood ratio chi-squared test produced a p-value indicating whether the simpler model was significantly worse than the previous model; this provided a second test to support the AIC comparison and also provided p-values for significance of model terms. The random effects accounted for the possibility that seeds were affected by some conditions particular to their dish and/or container, and were thus not truly independent replicates. They also accounted for temporal correlation produced by measuring the same unit over time. The significance of dish and container random effects was tested first; if significant then we included them in subsequent models to fully account for any possible pseudoreplication. If these tests indicated an overall difference between treatments, we then tested for differences between the three treatments. This was done by pooling the two most similar treatments, and testing whether this simplified model was better than the model with all treatments differentiated. Pooling was continued until all remaining differences were significant. For more specifics on the analysis, see Additional file 1: Table S1 and S2.

## Competing interests

The authors declare that they have no competing interests.

## Authors’ contributions

Both authors contributed to the design and development of the ideas within the study. MG conducted the study and MR ran the statistical analyses. MG wrote the manuscript and MR participated in the preparation of the manuscript. Both authors have read and approved this manuscript.

## Supplementary Material

Additional file 1: Table S1 Results of statistical analysis of basil germination data &**Table S2.** Results of statistical analysis of chilli germination data. Each step in the analyses is shown separately, starting with the maximal model, and showing how the model was simplified step by step. Click here for file
