# Supplementary material for: Love thy neighbour: facilitation through an alternative signalling modality in plants
Source: BMC Ecol. 2013 May 7;13:19. doi: 10.1186/1472-6785-13-19 (PMC3651341; doi:10.1186/1472-6785-13-19)
Supplement: Additional file 1: Table S1 — Results of statistical analysis of basil germination data &Table S2. Results of statistical analysis of chilli germination data. Each step in the analyses is shown separately, starting with the maximal model, and showing how the model was simplified step by step. [file 1472-6785-13-19-S1.pdf]

Additional file 1 [Gagliano and Renton]

Love thy neighbour: facilitation through an alternative signalling modality in plants

Table A1: Results of statistical analysis of basil germination data. Each step in the analysis is shown on a separate row, starting with the maximal model, and showing how the model was simplified step by step. Each row includes the name of the mixed effects model fitted at that step; all terms in the model (random effects shown in brackets); the resulting AIC for the fitted model; the candidate term to be dropped; the comparison model ('cf. mod', ie the more complex model containing the candidate term that was compared with the current model not containing the candidate term using a chi-squared test); the p-value obtained from the model comparison test; and the best model retained at the end of the step. Note that a significant p-value means that the simpler (current) model is significantly worse than the more complex comparison model, and so the more complex model is retained as 'best', while a non-significant p-value means that the simpler (current) model is not significantly worse than the more complex comparison model, and so the simpler model is retained as 'best'. Also note that in every case, the decision based on the p-value was supported by the AIC, such that the 'best' retained model always had the lower AIC.

name	all terms	df	AIC	candidate term	cf. mod	p-val	best
fm1	grp + t + (t | bx/dsh) + (1 | bx/dsh) + (t | bx) + (1 | bx) + grp:t	18	1831.6	-	-	-	-
fm2	grp + t + (t | bx/dsh) + (t | bx) + (1 | fm2: bx) + grp:t	16	1827.5	(1|bx/dsh)	fm1	1	fm2
fm3	grp + t + (t | bx) + (1 | bx) + grp:t	10	1931.0	(t|bx/dsh)	fm2	<0.0001***	fm2
fm4	grp + t + (t | bx/dsh) + (1 | bx) + grp:t	13	1821.5	(t|bx))	fm2	1	fm4
fm5	grp + t + (t | bx/dsh) + grp:t	12	1819.5	(1|bx)	fm4	0.9997	fm5
fm6	grp + t + grp:t	7	1937.6	(t|bx/dsh)	fm5	<0.0001***	fm5
fm7	grp + t + (t | bx/dsh)	10	1818.0	grp:t	fm6	0.2874	fm7
fm8	t + (t | bx/dsh)	8	1822.0	-grp	fm7	0.01841*	fm7
fm9	grp + (t | bx/dsh)	9	1884.7	t	fm7	<0.0001***	fm7
fm10~	newgrp + t + (t | bx/dsh)	9	1817.5	Open/Masked pooled	fm7	0.21	fm10
fm8+	t + (t | bx/dsh)	8	1822.0	All pooled	fm10	0.01129 *	fm10

~ at this step the Open and Masked treatments were pooled to test for whether there were significant differences between these two treatments; as p>0.05 and AIC was lower, the simpler model with pooled treatments was retained as 'best'

+ at this step the Control treatment was pooled with the previously pooled Control and Masked treatments to test for whether there were significant differences between the Open treatment and the others; as p<0.05 and AIC was higher, the more complex model with different treatments was retained as 'best'

Table A2: Results of statistical analysis of chilli germination data. Each step in the analysis is shown on a separate row, starting with the maximal model, and showing how the model was simplified step by step. Each row includes the name of the mixed effects model fitted at that step; all terms in the model (random effects shown in brackets); the resulting AIC for the fitted model; the candidate term to be dropped; the comparison model ('cf. mod', ie the more complex model containing the candidate term that was compared with the current model not containing the candidate term using a chi-squared test); the p-value obtained from the model comparison test; and the best model retained at the end of the step. Note that a significant p-value means that the simpler (current) model is significantly worse than the more complex comparison model, and so the more complex model is retained as 'best', while a non-significant p-value means that the simpler (current) model is not significantly worse than the more complex comparison model, and so the simpler model is retained as 'best'. Also note that in every case, the decision based on the p-value was supported by the AIC, such that the 'best' retained model always had the lower AIC.

name	all terms	df	AIC	candidate term	cf. mod	p-val	best
fm1	grp * t + (t | bx/dsh) + (1 | bx/dsh) + (t | bx) + (1 | bx)	18	1297.0	-	-	-	-
fm2	grp + t + (t | bx/dsh) + (t | bx) + (1 | fm2: bx) + grp:t	16	1293.4	(1|bx/dsh)	fm1	1	fm2
fm3	grp + t + (t | bx) + (1 | bx) + grp:t	10	1381.1	(t|bx/dsh)	fm2	<0.0001***	fm2
fm4	grp + t + (t | bx/dsh) + (1 | bx) + grp:t	13	1287.4	(t|bx))	fm2	1	fm4
fm5	grp + t + (t | bx/dsh) + grp:t	12	1285.4	(1|bx)	fm4	0.9997	fm5
fm6	grp + t + grp:t	7	1398.3	(t|bx/dsh)	fm5	<0.0001***	fm5
fm7	grp + t + (t | bx/dsh)	10	1282.2	grp:t	fm6	0.6765	fm7
fm8	t + (t | bx/dsh)	8	1284.6	-grp	fm7	0.04064*	fm7
fm9	grp + (t | bx/dsh)	9	1342.5	t	fm7	<0.0001***	fm7
fm10~	newgrp + t + (t | bx/dsh)	9	1280.2	Control/Masked pooled	fm7	0.9037	fm10
fm8+	t + (t | bx/dsh)	8	1284.6	All pooled	fm10	0.01147 *	fm10

~ at this step the Control and Masked treatments were pooled to test for whether there were significant differences between these two treatments; as p>0.05 and AIC was lower, the simpler model with pooled treatments was retained as 'best'

+ at this step the Open treatment was pooled with the previously pooled Control and Masked treatments to test for whether there were significant differences between the Open treatment and the others; as p<0.05 and AIC was higher, the more complex model with different treatments was retained as 'best'
